# Selection of the Anti‐Osteoporosis Active Ingredients of Fructus Psoraleae—Eucommia—Drynariae Rhizoma Based on Solid‐Phase Bio‐Cell Chromatography and HPLC–MS Analysis

**DOI:** 10.1002/fsn3.4604

**Published:** 2025-01-14

**Authors:** Liming Zhu, Yeqing Wang, Zhongxin Zhu, Xiaocong Yao, Ruijuan Zhang, Yujie Xia, Minbo Liu

**Affiliations:** ^1^ Department of Osteoporosis Care and Control Xiaoshan Affiliated Hospital of Wenzhou Medical University Zhejiang Hangzhou China; ^2^ Department of Pharmacy Xiaoshan Affiliated Hospital of Wenzhou Medical University Zhejiang Hangzhou China; ^3^ Department of Clinical Research Center Xiaoshan Affiliated Hospital of Wenzhou Medical University Zhejiang Hangzhou China; ^4^ Department of Research and Development Zhejiang Zhongwei Medical Research Center Zhejiang Hangzhou China

**Keywords:** anti‐osteoporosis active ingredients, Fructus Psoraleae—Eucommia—Drynariae Rhizoma, osteoporosis, solid‐phase bio‐cell chromatography and HPLC–MS

## Abstract

Osteoporosis (OP) is a prevalent metabolic bone disease globally. Currently, the development of Traditional Chinese Medicine (TCM) resources to unblock joints, strengthen bones, and enhance muscle function to regulate anti‐osteogenic and anabolic metabolism and thus reshape intraosseous homeostasis was an effective way to alleviate OP. The F–E–D formula, comprising Fructus Psoraleae, Eucommia, and Drynariae Rhizoma, has shown efficacy in treating OP. However, its complex natural components necessitate the screening and simplification of bioactive compounds to further elucidate their therapeutic mechanisms and enhance therapeutic efficacy. In this study, we first used drug–target binding to produce different effects, which in turn exhibited different retention characteristics on the stationary phase. Using osteoblasts and osteoclasts as stationary phases, a chromatographic system (Solid‐phase Bio‐cell Chromatography, SBC) had been constructed to mimic the drug–target interaction, and the separation, analysis, and bioactivity screening of the chemical components of F–E–D had been performed. Then, the above collected eluates were analyzed by fine metabolomics, and 95 effective metabolites were initially screened and combined with database screening to finally select betaine, L‐fucose, and itaconic acid as potentially active candidate compound monomers for the interaction with osteoblast–osteoclast in F–E–D. In terms of cell validation experiments, we found that the screened active monomers significantly inhibited the formation of osteoclasts, and the itaconic acid–treated group played a significant inhibitory effect on the expression of inflammatory factors TNF‐α and IL‐6. The above experimental data showed that the monomeric active ingredients in TCM could be effectively screened by solid‐phase bio‐chromatography and HPLC–MS, and the in vitro cellular experiments verified that the active monomers of TCM slowed down the progression of OP by inhibiting osteoclast production and alleviating the expression of inflammation.

## Graphical abstract

In this study, we employed solid‐phase bio‐chromatography and HPLC–MS to screen the monomeric active ingredients in the three minor formulas (Fructus Psoraleae—Eucommia—Drynariae Rhizoma, F–E–D). Then, we investigated that the active monomers slowed down the progression of OP by inhibiting osteoclast production and alleviating the expression of inflammation by in vitro cellular experiments.

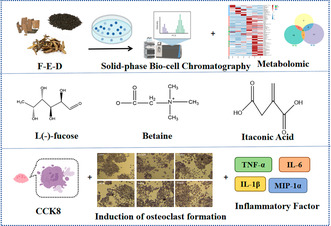

## 1 Introduction

Osteoporosis (OP) was the most common systemic metabolic bone disease in the world, which featured with microarchitectural deterioration, reduced bone mass, and increased fracture risk (Ye et al. 2020). At present, with about 6000w patients in China, and the incidence of OP increased with age, especially postmenopausal women, around one‐third of them would suffer OP (Dewan et al. 2018) (Zeng et al. 2019). Owing to its insidious pathogenesis, the symptoms of OP generally did not become apparent until a fracture occurs, thus posing a serious threat to the patient's health. When OP happened, it would lead to a decrease in the quality of life, often with symptoms such as back pain, leg cramps, etc. If the patient had a fall, it would lead to fractures of the thoracic spine, lumbar spine, distal radius, femoral neck, and proximal humerus (Compston, McClung, and Leslie 2019). Recent studies had found that the essence of OP occurred due to the imbalance of bone balance in the body, which indicated that when the rate of bone resorption was faster than the rate of bone formation, the bone homeostasis would be disrupted (Huang et al. 2023; Lerner, Kindstedt, and Lundberg 2019). Based on the multiple regulatory mechanisms of bone homeostasis, a series of drugs targeting OP were emerged in clinical practice, including bisphosphonates, selective estrogen receptor modulators, calcitonin, and molecularly targeted drugs (Awasthi et al. 2018). However, there were some adverse effects associated with the long‐term use of these drugs, such as an increased risk of cancer, stroke, bone necrosis, and disruption of body metabolism (Frith et al. 2018; Tański, Kosiorowska, and Szymańska‐Chabowska 2021). Therefore, researchers are focusing on finding drugs with the same effect, among which traditional Chinese medicine (TCM) as a mild analogue of chemical drugs has become a research hotspot.

Compared to the current clinically used anti‐OP chemical drugs, which had many negative effects in application, TCM could not only promote bone formation and diminish unbalanced bone resorption but also improve bone density and biomechanical properties and reduce bone microstructure degradation, TCM also played an anti‐metabolic and anabolic role in bone metabolism (He et al. 2019; Peng, Xu, and You 2022). TCM had been widely used in the treatment of OP since ancient times, such as kidney‐supplementing blood‐quickening decoction, kidney nourishing and bone strengthening soup, nameless iso‐punch, and bone protector combination, etc. (He et al. 2019; Peng, Xu, and You 2022; Zhuo, Li et al. 2022). Among them, Fructus Psoraleae, Eucommia, and Drynariae Rhizoma were the representative drugs for strengthening bones in the above‐mentioned remedies (Han et al. 2022; Liu et al. 2020). It had a long history of application. Based on the theory of Chinese medicine, it was found that the growth of bones depends on the nourishment of kidney essence, and these herbs have Wenshenyang, tonifying the kidney and strengthening the bones (Xin et al. 2019). In the clinical practice of OP, those drugs also showed good efficacy; they could provide a certain complement and modulation to the chemical OP therapy, with the effect of inhibiting osteoclasts, promoting osteogenesis, and improving bone loss in ovariectomized mice (Chen et al. 2020). In recent years, combined with the basic pathogenesis of primary OP back pain, Chinese medicine experts had proposed to synthesize three small formulas for OP treatment by selecting Fructus Psoraleae, Eucommia, and Drynariae Rhizoma. However, the actual clinical use of the above‐mentioned F–E–D drug was mainly through simple decoction of the compound drug, but the natural drug composition was complex, and there were a large number of ineffective or even harmful ingredients in the soup. For example, the discovered toxicity of Fructus Psoraleae mainly included hepatotoxicity, nephrotoxicity, phototoxicity, developmental toxicity, and reproductive toxicity, among which hepatotoxicity is most predominant, and psoralen, isopsoralen, bavachinin A, and bakuchiol were the main toxic components (Xia et al. 2018) (Zhuo, Jing et al. 2022). The major constituents of *Eucommia ulmoides* such as lignans, flavonoids, and iridoids have poor oral bioavailability (Liang et al. 2017). Drynariae Rhizoma contained naringin, which could lead to toxicity and damage the organs and brain when ingested in excessive amounts (Ahn et al. 2024). Therefore, if we wanted to develop the F–E–D, we needed to further elucidate its bioactive components and its therapeutic mechanism, which was one of the key issues limiting its development at present.

In TCM research, the traditional approach for screening bioactive compounds in TCM was the isolation and purification of their individual or single class components, followed by in vivo or in vitro bioactivity evaluation. This approach was time‐consuming and inefficient. Especially when the content of a component in herbal medicine was low, its isolation and purification were very difficult and required high cost (Wang et al. 2016). In order to solve this key problem that has restricted the basic research on the efficacy substances of TCM, cellular bio‐chromatography has been developed vigorously. As a new chromatographic technique, cellular bio‐chromatography was developed to analyze the effector substances in TCM and its compounds by using the principle of drug binding to the targets (enzymes and receptors) to produce effects (C. Chen et al. 2013). By directly selecting cells of the drug effector organ as the stationary phase, a chromatographic system that mimics drug–target interactions has been formed. For instance, Yang et al. (2018) used biological cell chromatography combined with GC histological analysis with the aid of cells to explore the active substances in the Chinese medicine Tongkang Huanhe Tang (TQHXD) and found that the pretreatment of PC12 cells with muscarinone significantly increased cell viability and reduced lactate dehydrogenase (LDH) release and apoptosis. Combined with the pharmacodynamic study, musk ketone was identified as one of the active ingredients of TQHXD. Biocellular chromatography utilized the fact that different components of TCM had different binding abilities to their targets and exhibited different retention characteristics on the stationary phase, thus realizing the simultaneous separation and analysis of chemical components of TCM and biological activity screening, and its efficiency was greatly improved compared with the traditional methods.

In view of the above‐mentioned, the current research on the active substances of TCM was mainly focused on the monomeric components such as Fructus Psoraleae, Eucommia, Drynariae Rhizoma, and Phellodendron, but the research on the specific active ingredients of traditional small formulae had not been studied systematically. Therefore, the aim of this study was to identify the anti‐OP bioactive components in this compound by solid‐phase osteoblast–osteoclast chromatographic screening of F–E–D by cellular bio‐chromatography combined with liquid‐mass spectrometry. This study would provide an experimental basis for the clinical treatment of OP by F–E–D, as well as a theoretical basis for the further development of OP agents and the simplification of compound TCMs.

## 2 Materials and Methods

### 2.1 Chemicals and Materials

Fructus Psoraleae, Eucommia, and Drynariae Rhizoma (Bozhou Huaishun Tang, Anhui Province); acetonitrile (chromatographic grade, Maclean's reagent); methanol (chromatographic grade, Maclean's reagent); formic acid (AR, Maclean's reagent); acetic acid (AR, Maclean's reagent); DMEM high sugar medium (Biosharp); α‐MEM medium (Cytiva); Fbovine serum (VivaCell); trypsin (Sigma); PBS phosphate buffer (Biosharp); penicillin and streptomycin mixture (Beyotime); RAW264.7 cells (Shanghai Cell Bank, Chinese Academy of Sciences); MC3T3‐E1 Subclone14 (Shanghai Cell Bank, Chinese Academy of Sciences); betaine, itaconic acid, and L‐fucose (www.bzwz.com); LPS and anti‐tartrate acid phosphatase staining (Solaibao); RNA pure Tissue & Cell Kit (DNase I); HiFi Script cDNA Synthesis Kit, Magic SYBR Mixture (Kangwei Century); primers β‐actin, IL‐6, TNF‐α, MIP‐1α, and IL‐1β (Shanghai Biotech Co. Ltd.); and CCK‐8 kit.

### 2.2 Fructus Psoraleae—Eucommia—Drynariae Rhizoma Extract

Fructus Psoraleae, Eucommia, and Drynariae Rhizoma were ground and passed through 100 mesh sieves. Then, 1 g of the crushed drug powder was weighed, and 10 mL of ultrapure water was added with 60°C, 150w ultrasonic extraction for 30 min. Finally, it was passed through a 0.22‐um filter membrane and set aside.

### 2.3 Cell Culture

RAW264.7 cells were removed from liquid nitrogen and resuscitated with DMEM high sugar medium containing 1% penicillin and 10% FBS, incubated at 37°C in a 5% CO_2_ constant temperature incubator and passaged once every 2 d. The cells were allowed to rejuvenate for subsequent experiments. The above cultivated RAW264.7 cells were inoculated in 6‐well plates and induced with 1 μg/mL of LPS after 24 h of cell wall attachment. LSP concentrations were referenced to the method of Cao et al. (2019). After 24 h of induction, the medium was discarded and replaced with a new medium and incubated for 4 days. The cells were fixed in 4% paraformaldehyde after Day 5. The TRAP staining solution was configured according to the instructions, incubated for 1 h at 37°C in a biological incubator protected from light, rinsed well with alkaline solution, air‐dried, observed, and counted under a light microscope.

The above induced osteoblasts with good status were selected, the culture medium was poured off, and the serum‐free culture medium was added and incubated at 37°C and 5% CO_2_ for 0.5 h. Then, the culture medium was poured off and 1 mL of drug extract was added, and 1 mL of serum‐free culture medium was added to the control group; then, the drug solution was poured off after continuing incubation for 1 h, and the cells were washed with 2 mL of PBS 5 times, and the last eluate was collected. The washed cells were repeatedly freeze–thawed three times at −80°C, sonicated and broken by adding 10 mL of ethanol, and the supernatant was removed by centrifugation at 10,000 rpm for 10 min, volatilized under reduced pressure, and lysed by adding 1 mL of distilled water and set aside. The last resolubilized cell lysate was subjected to HPLC and HPLC–MS analysis.

MC3T3‐E1 cells were removed from liquid nitrogen and resuscitated with α‐MEM containing 1% penicillin and 10% FBS, incubated at 37°C in a 5% CO_2_ constant temperature incubator and passaged once every 2 d. The cells were allowed to rejuvenate for subsequent experiments. Then, the above induced MC3T3‐E1 cells with good status were selected and followed the osteoclast dosing steps above. Finally, the last resolubilized cell lysate was subjected to HPLC and HPLC–MS analyses.

### 2.4 Chromatography and Mass Spectrometry Conditions

Chromatography conditions: The gradient elution was performed on an ACQUITY UPLC HSS T3 1.8 μm (2.1 × 150 mm) column with an autosampler set at 8°C. 2 μL was injected at a flow rate of 0.25 mL/min and a column temperature of 40°C. The mobile phase was positive ion 0.1% formic acid in water (C)—0.1% formic acid in acetonitrile (D); negative ion 5 mM ammonium formate in water (A)—acetonitrile (B). The gradient elution program was 0 ~ 1 min, 2% B/D; 1–9 min, 2%–50% B/D; 9 ~ 12 min, 50%–98% B/D; 12–13.5 min, 98% B/D; 13.5–14 min, 98%–2% B/D; 14–20 min, 2% D—positive mode (14–17 min, 2% B—negative mode).

Mass spectrometry conditions: The instrument uses an electrospray ionization source (ESI) in positive and negative ionization modes with a positive ion spray voltage of 3.50 kV and a negative ion spray voltage of 2.50 kV, a sheath gas of 30 arb, an auxiliary gas of 10 arb, a capillary temperature of 325°C, full scan at a resolution of 70,000, a scan range of 81–1000, and secondary cleavage using HCD with a collision voltage of 30 eV, while dynamic exclusion was used to remove unnecessary MS/MS information.

### 2.5 Active Substance Data Selection

The above mass spectrometry data (see Supporting Information S1) were first calculated as the ratio of each monomer sample/blank, arranged in descending order, screened for monomers with a ratio greater than or equal to 5.0, then removed the prevalent and common components, and filtered by literature search with the keywords bone, OP, osteoblast, and osteoclast, and intersected with these keywords. The monomers that intersected with these keywords were selected as the active substances for the next step of validation.

### 2.6 Active Substance Verification

RAW264.7 was induced into osteoclasts according to the method in Section 2.3, and the induced osteoclasts were divided according to the control group, inflammation model group, and different concentrations of the drug group, and the monomers screened in Section 2.5 were added for drug culture for relevant experiments. The concentration of betaine (0, 125, 250, 500, and 1000 μM) was referenced to the Yajun et al. (2021) method, the concentration of itaconic acid (0, 2, 4, 6, and 8 mM) was referenced to the Qu et al. (2021) method, and the concentration of L‐fucose (0, 12.5, 25, 50, and 100 μg/mL) was referenced to the Zhu et al. (2022) method.

### 2.7 CCK8

Cytotoxicity assay: RAW264.7 cells were divided into the control group, inflammation model group, and different concentrations of drug group, and inoculated in 96‐well plates according to 1 × 10^4^ cells per well, and 5 replicate wells were set for each group. Control group cells were cultured for 24 h and then replaced with a DMEM high sugar medium for 24 h. Inflammation model group cells were cultured for 24 h with a pre‐experimentally optimized lipopolysaccharide (LPS) concentration and then replaced with a DMEM high sugar medium for 24 h. And drug administration group cells were induced with LPS for 24 h and then replaced with a DMEM high sugar medium containing a certain concentration gradient of drug for 24 h. All groups discarded the medium, and 100 μL of DMEM medium and 10 μL CCK‐8 reagent were added to each well, and the incubation was continued for 1 h; the absorbance (A) value at a wavelength of 450 nm was measured by the enzyme standardization instrument.

### 2.8 TRAP Staining

RAW264.7 cells were inoculated with 1 × 10^4^ cells/well in a 6‐well plate and cultured for 12 h. After the cells were plastered, they were grouped and treated as follows. The control group was cultured for 5 days with daily fluid changes. The LPS induction group was induced with LPS for 24 h and then cultured for 4 days with daily fluid changes. And the drug administration group was induced with LPS for 24 h and then replaced with a DMEM complete medium containing a certain concentration of drug for 24 h and discarded. The medium was replaced with a DMEM complete medium for 3 days, and the liquid was changed every day. The cells were fixed with 4% paraformaldehyde after the 5th day. The TRAP staining solution was configured according to the instructions, incubated for 1 h at 37°C in a biological incubator protected from light, rinsed well with alkaline solution, air‐dried, observed, and counted under a light microscope.

### 2.9 Q‐PCR

Cells were induced by LSP (1 μg/mL) for 22 h (Qin et al. 2018), and Q‐PCR was determined according to the subordinate manner. For RNA extraction, RNA was extracted according to the instructions of RNA pure Tissue & Cell Kit (DNase I) (Kangwei Century). Reverse transcription was performed according to the instructions of HiFi Script cDNA Synthesis Kit (Kangwei Century) to reverse transcribe RNA to cDNA. Q‐PCR was performed according to the instructions of Kangwei Century The Q‐PCR assay was performed according to the instructions of Kangwei Century. Q‐PCR reactions were performed for IL‐6, TNF‐α, MIP‐1α, and IL‐1β. In this experiment, β‐actin was used as the internal reference (Table 1).

**TABLE 1:** Fluorescent quantitative primers.

Name	Sequence (5′–3′)
β‐Actin‐F	TGT CCA CCT TCC AGC AGA TGT
β‐Actin‐R	AGC TCA GTA ACA GTC CGC CTA GA
IL‐6‐F	TGG GAC TGA TGC TGG TGA CA
IL‐6‐R	ACA GGT CTG TTG GGA GTG GT
IL‐1β‐F	TTG ACG GAC CCC AAA AGA TG
IL‐1β‐R	AGA AGG TGC TCA TGT CCT CA
TNF‐α‐F	ATG GAT CTC AAA GAC AAC CAA CTA G
TNF‐α‐R	ACG GCA GAG AGG AGG TTG ACT T
MIP‐1α‐F	CAA GCA GCA GCG AGT ACC AGT C
MIP‐1α‐R	GAG AAG AAC AGC AAG GGC AGT GG

### 2.10 Statistical Analysis

All data were processed by GraphPad Prism8 and statistically analyzed using one‐way ANOVA, and the values of *p* < 0.05 were considered statistically significant.

## 3 Results and Discussion

In order to investigate the anti‐OP active substances in Fructus Psoraleae—Eucommia—Drynariae Rhizoma, this experiment was first conducted by liquid chromatography analysis of the last eluate (blank group) and the resolubilized cell lysate (sample group) of the three monomers through osteogenic and osteoclastic experiments. And the primary screening of active substances was done with the help of metabolomics. Then, cellular experiments were used to verify the effect of the above primary screening active substances on osteoclasts.

### 3.1 Solid‐Phase Bio‐Cell Chromatography for Primary Screening of the Active Substances From F–E–D

**FIGURE 1:**
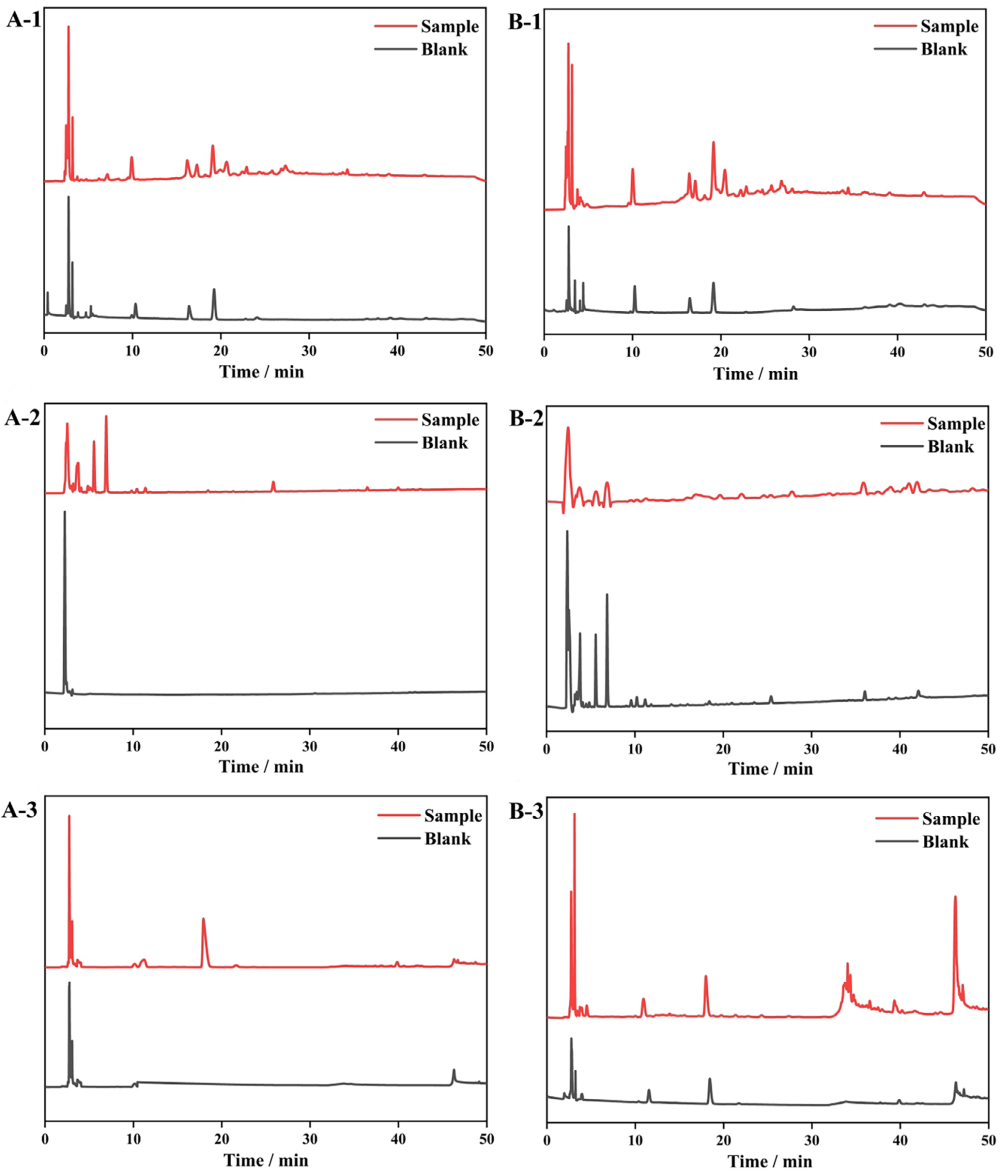
Solid‐phase bio‐cell chromatography results of F–E–D. A‐1, B‐1. The spectra of Drynariae Rhizoma in osteoblasts and osteoclasts as stationary phases. A‐2 and B‐2. The spectra of Fructus Psoraleae in osteoblasts and osteoclasts as stationary phases. A‐3 and B‐3. The spectra of Eucommia in osteoblasts and osteoclasts as stationary phases.

**FIGURE 2:**
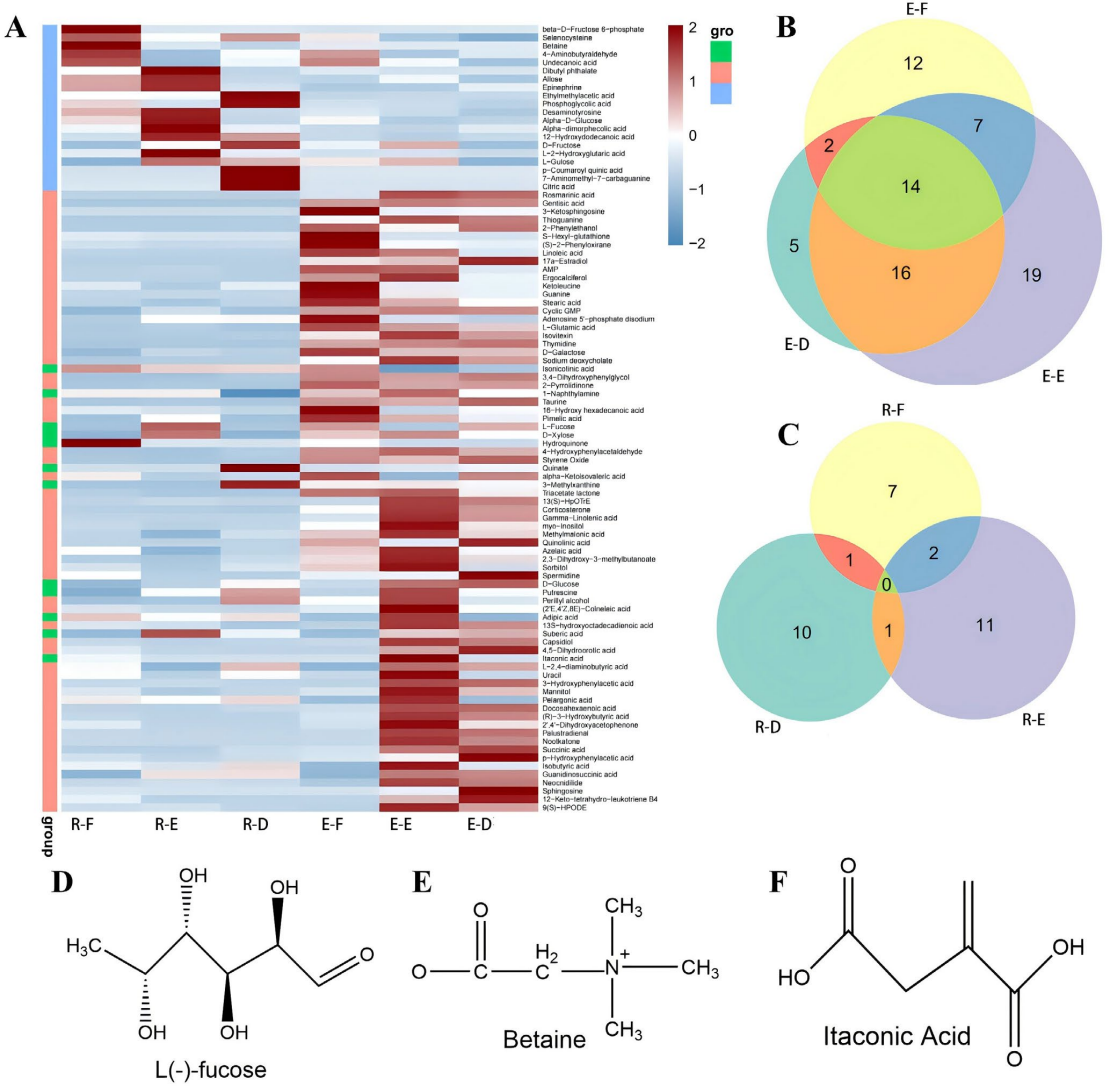
Metabolomic screen for active substances in F–E–D. A. Heat map of metabolite differences in osteoblasts and osteoclasts. B. Venn diagram of metabolite differences in osteoblasts. C. Venn diagram of metabolite differences in osteoclasts. D. L‐fucose chemical formula. E. Betaine chemical formula. F. Itaconic acid chemical formula. “E–F,” “E–E,” and “E–D” stand for MC3T3‐E1 cells incubated with Fructus Psoraleae, Eucommia, and Drynariae Rhizoma, respectively. As in Figure 2.C, “R–F," “R–E,” and “R–D" stand for RAW264.7 cells incubated with Fructus Psoraleae, Eucommia, and Drynariae Rhizoma, respectively.

**FIGURE 3:**
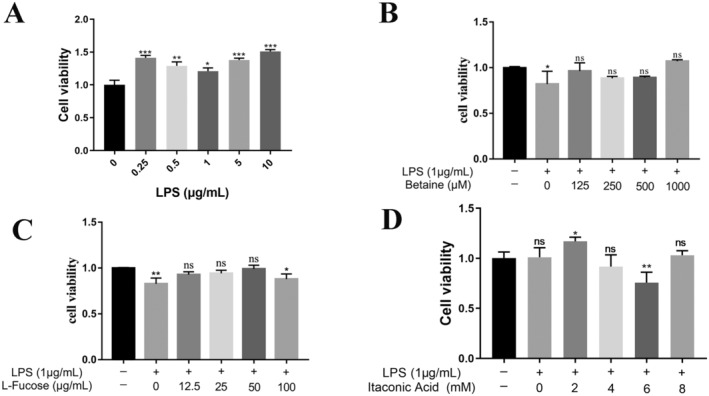
In vitro cytotoxicity results of active monomeric substances. A. CCK‐8 assay of RAW264.7 cytotoxicity induced by different concentrations of LPS. B. CCK‐8 assay of RAW264.7 cytotoxicity induced by betaine on 1 μg/mL of LPS. C. CCK‐8 assay of RAW264.7 cytotoxicity induced by L‐fucose on 1 μg/mL of LPS. D. CCK‐8 assay of RAW264.7 cytotoxicity induced by itaconic acid on 1 μg/mL of lipopolysaccharide. *p < 0.05 as denoted by a bar, **p < 0.01 as denoted by a bar, ***p < 0.001 as denoted by a bar.

Firstly, the crude sample crushing was passed through 100 mesh sieves according to the monomeric components, and then the powder was passed through 95% ethanol and petroleum ether in turn for crude extraction, and the extract was obtained by using a rotary evaporator, and then the extract solution was incubated into osteocytes and osteoblasts. After incubation, the unbound components were eluted, the bound components were dissociated, and the remaining cells were lysed to obtain the lysate. The last eluate and the resolubilized cell lysate were subjected to HPLC, and the results at 230 nm are shown in Figure 1. The differences in the peak time and peak area of the active substances in the extracts were explored by using osteoblasts and osteoclasts as stationary phases, and the differences were a reflection of the combination of different active substances with osteoblasts and osteoclasts in these three herbal medicines. From Figure 1, three kinds of herbal medicines were found to show different trends on the binding effect of osteoblasts and osteoclasts. Figures 1A–1 and B‐1 show the results of incubation of osteoblasts and osteoclasts on the *Drynariae Rhizoma* crushing tonic extract. It could be found that some different peaks appeared in the administered group within 20–40 min, which indicated that the active substances in the *Drynariae Rhizoma* crushing tonic extract were combined with osteoclasts and osteoclasts to produce the effect and showed different retention characteristics. Figures 1, 2 and 1, 2 show the results of osteoblast and osteoclast incubation with *Fructus Psoraleae* extracts. From the figures, it was found that the *Fructus Psoraleae* extract binds more readily to the osteoclasts, and its characteristic peaks presented in the osteoclast phase were more pronounced than in the osteoclasts. Figures 1, 2, 3 and 1, 2, 3 show the results of incubation of osteoblasts and osteoclasts on the Eucommia extract. A clear difference between the targets of Eucommia extract combined with osteoblasts and osteoclasts was presented in the time and location of peak emergence.

The main active monomers of *Drynariae Rhizoma* were flavonoids, triterpenoids, phenylpropanoids, and lignans, which had the effect on anti‐OP and promoting fracture healing (Chen et al. 2021). Qiao et al. (2014) analyzed the active substances of *Drynariae Rhizoma* by the liquid phase and found that the active substances of bonesetter were mainly flavonoids, anthocyanins, and triterpenoids at 20–40 min after elution and purification. Therefore, we speculated that the active substances of osteoclasts and osteoblasts in this experiment could be the same substances mentioned above, but the specific active substances would be investigated further with the help of metabolomics. Similarly, *Fructus Psoraleae* was also a herbal medicine commonly used in the treatment of OP, and its main active substances were found to be coumarin, psoralen, iso‐psoralen, psoralen, and iso‐psoralen in the previous study (Yang et al. 2018). Khuranna et al. (2020) analyzed the active substances of *Fructus Psoraleae* under different isolation conditions and found that the extracts extracted using ethanol and petroleum ether were the most protective of the active substances in bone tonic, and with the help of HPLC, it was found that the active substances in the extracted bone tonic also appeared within 20–30 min, which was consistent with our experimental results, and this indicated that we successfully extracted the active substances of bone tonic through ethanol and petroleum ether and embodied them using biocellular chromatography. The aqueous solution of Eucommia (EU), a herbal medicine commonly used in the treatment of cardiovascular diseases, orthopedics, cancer, metabolic syndrome, and neurological disorders (Hussain et al. 2016). The main active substances found in its aqueous solution were myostatin and isoquercitrin, including rosmarinic acid, kynylopodic acid, rutin, verbascoside, chlorogenic acid, and astragaloside. The location of the peak of Eucommia tulipata in this experiment at 20–40 min was similar to Guan et al.'s study (Guan et al. 2021), which found that the substances at that point were chlorogenic acid and astragaloside and other substances. Generally speaking, the active substances of the three minor formulas were found to be eluted and combined with the cells by biological cell chromatography against the lysates of osteoblasts and osteoclasts, in turn, which realized the simultaneous separation and analysis of the chemical components of TCMs, but the specific related substances were further explored in the subsequent metabolomics.

### 3.2 Metabolomics for the Identification of the Active Substances in the F–D–E

In order to investigate the above‐mentioned separation of the specific substances in the three minor formulae of Fructus Psoraleae—Eucommia—Drynariae Rhizoma based on bio‐cell chromatography, the above collected cell lysates were analyzed by HPLC–MS, while the obtained data were analyzed bioinformatically. The data differences of bioinformatics are shown in Figure 2, and the specific metabolized substances are shown in Appendix S1. Figure 2A shows a heat map difference analysis of metabolites from Fructus Psoraleae—Eucommia—Drynariae Rhizoma on osteoblast and osteoclast lysates after screening them through the database. In the cross‐sectional comparison of Figure 2A, it was found that the active substances in the three minor formulae were classified as acting on osteoclasts in smaller amounts than osteoblasts, and the main differences in such active substances were concentrated in the osteoclast piece. Metabolites such as isonicotinic acid, itaconic acid, L‐fucose, 1‐naphthylamine, D‐xylose, hydroquinone, 4‐hydroxyphenylacetaldehyde, and quinic acid are found in both osteoblast and osteoclast metabolites. The metabolites attributed to osteoblasts in the three minor formulae were found to be more pronounced in osteoclasts derived from Eucommia and Drynariae Rhizoma. The cross‐sectional data analysis revealed that most of the differences in the active substances in the three minor formulae were mainly in osteoblasts, and these metabolites could improve OP by inhibiting osteoclasts, in turn. In the longitudinal data, metabolites were evident in osteoblasts than in osteoclasts, especially in Eucommia and Drynariae Rhizoma where metabolites were more diverse. Figure 2B shows the metabolic differences in osteoblasts by the Venn diagram, in which there were 56, 37, and 35 metabolites of Eucommia, Drynariae Rhizoma, and Fructus Psoraleae, respectively, with 14 metabolites of common active substances acting on osteoclasts in these three Chinese herbs. In Figure 2C, the metabolic differences in osteoclasts were shown by the Venn diagram, in which there were 14, 12, and 10 metabolites of Eucommia, Drynariae Rhizoma, and Fructus Psoraleae, respectively, with no common metabolites acting on osteoblasts in the three minor herbal remedies. The comparison of heat map and Venn diagram showed that the active substances in the three minor formulae acted on osteoblasts more concentrated and showed diversity.

The essence of OP in bone was an imbalance in bone homeostasis (Song et al. 2022), and reestablishing the balance of bone homeostasis had become a hot spot for research to improve OP. Intrabody homeostasis was essentially the maintenance of a proper balance between bone resorption by osteoclasts (OCs) and bone matrix formation by osteoblasts (OBs) through a series of complex and closely regulated processes, and promoting outcome cells by inhibiting osteoclasts would be an effective way to improve OP (Kenkre and Bassett 2018). Eucommia, Drynariae Rhizoma, and Fructus Psoraleae were TCMs that had been shown to promote OP repair, but the specific mechanism of their specific improvement in OP was unknown due to the significant biological activity using multicomponent drugs and the ability to act on multiple targets through multiple components. Metabolic bioinformatics has shown potential in bioactivity assessment and mechanism of actions for herbal medicines, as well as in drug research and development, based on the holistic metabolic profiles in complex biological matrices, which provided variants of systematic metabolic networks, as a holistic study of multiple metabolic responses to pathological stimuli and drug treatments in complex biological systems. (Wang et al. 2017). Liang et al. investigated the possible pharmacological effects of the Chinese herbal medicine *Ginkgo biloba* (GBCCM) at different doses or ratios by using bone marrow mesenchymal stem cells (BMSCs) combined with metabolomics, and found that eight compounds in GBCCM were involved in body metabolism, where GBCCM mainly affected the signaling pathways of unsaturated fatty acids, pyruvate, bile acids, melanin, and stem cells, among which flavonoid glycosides (FAs) and terpene lactones (TLs) had the effects of lowering blood pressure and regulating stem cell proliferation and melanogenesis (Liang et al. 2023). Metabolite bioinformatics has simplified the screening of active substances in TCM while providing a reference for the drug‐forming properties of TCM. In this experiment, we selected osteoclasts and osteoblasts to investigate the active ingredients in the three minor formulas and found a total of 260 metabolites through screening. A total of 95 metabolites were screened by calculating the ratio of each monomer sample/blank and arranging them in descending order, and the monomers with a ratio greater than or equal to 5.0 were then subjected to heat map and Venn diagram analysis. The active substances in the minor formula were found to alleviate OP mainly by inhibiting osteoclasts. We first removed the prevalent and common components, then filtered the monomers that intersected with these keywords by literature search with the keywords bone, OP, osteoblast, and osteoclast, and finally selected itaconic acid, L‐fucose, and betaine as the validation objects (Figure 2D–F). Combined with the previous heat map and Venn diagram data, osteoclast was chosen as an example to validate its anti‐OP ability.

### 3.3 Osteoclast Validation Experiments With Active Monomeric Substances

Based on the screening results of the above histological information, itaconic acid, L‐fucose, and betaine were selected as validation targets. The effects of LPS, itaconic acid, L‐fucose, and betaine on the proliferation activity of RAW264.7 cells were investigated at the cell number level by the CCK‐8 method. The effect of active monomeric substances on osteoclast formation was then investigated by staining and counting.

**FIGURE 4:**
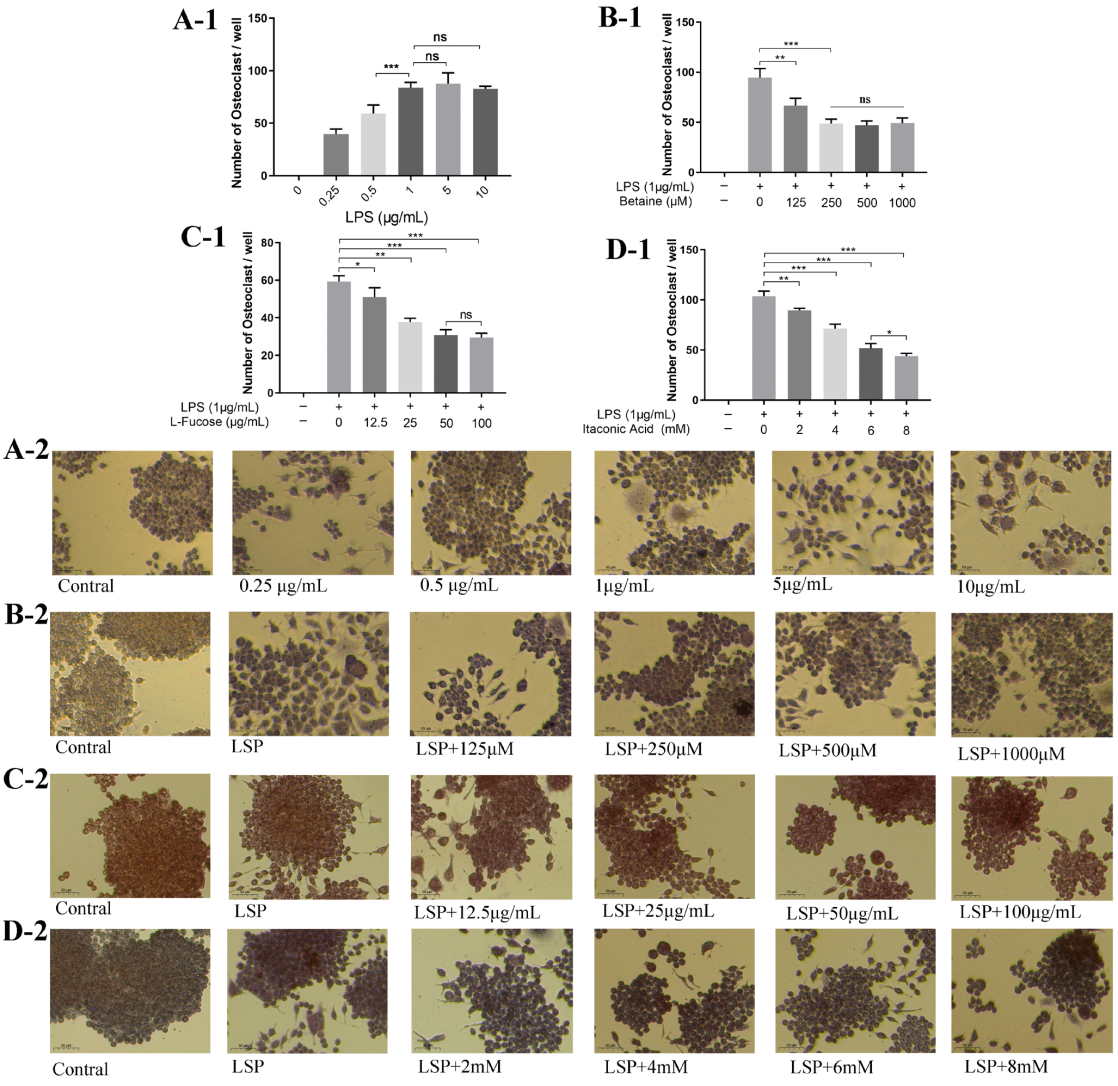
Results of the induction of osteoclast formation by active monomeric substances in vitro. A‐1. Number of osteoclasts induced by different concentrations of LPS. B‐1. Effect of different concentrations of betaine on the number of osteoclasts formed by 1 μg/mL of LPS‐induced RAW264.7. C‐1. Effect of different concentrations of L‐fucose on the number of osteoclasts formed by 1 μg/mL of LPS‐induced RAW264.7. D‐1. Effect of different concentrations of itaconic acid on the number of osteoclast formation induced by 1 μg/mL of LPS RAW264.7. A‐2. TRAP staining of osteoclasts under LPS induction (×20). B‐2. TRAP staining of osteoclasts under betaine incubation (×20). C‐2. TRAP staining of osteoclasts under L‐fucose incubation (×20). D‐2. TRAP staining of osteoclasts under itaconic acid incubation (×20). *p < 0.05 as denoted by a bar, **p < 0.01 as denoted by a bar, ***p < 0.001 as denoted by a bar.

Figure 3 shows the effect of LPS and screened active substances on the proliferative activity of RAW264.7 cells. In Figure 3A, different concentrations of LPS (0, 0.125, 0.25, 0.5, 1, 5, 10 μg/mL) were induced for 24 h and then incubated with the CCK8 reagent for 4 h at 37°C. It was found that different concentrations of LPS had a proliferative effect on RAW264.7 cells, with 1 μg/mL having the least effect on cell activity. The effects of betaine (0, 125, 250, 500, and 1000 μM), L‐fucose (0, 12.5, 25, 50, and 100 μg/mL), and itaconic acid (0, 2, 4, 6, and 8 mM) on the LSP‐induced cellular activity of RAW264.7 were also assayed according to the same steps as described above, and in Figure 3B, betaine concentrations of 125 to 1000 μM had no effect. In Figure 3C, none of the cellular activities of RAW264.7 were affected by the concentration of itaconic acid from 0 to 8 mM. In Figure 3D, all cellular activities of RAW264.7 were not affected when the concentration of rockulose was 12.5–50 μg/mL. When the concentration of rockulose was 100 μg/mL, there was an inhibitory effect on the cellular activity of RAW264.7. The effect of the screened monomers on osteoclast formation was further explored after cytotoxicity. The ability of LPS at different concentrations to induce RAW264.7 cells into osteoblasts was firstly investigated, the morphology of RAW264.7 cells differentiated into osteoblasts was observed by TRAP staining, and the specific experimental results on osteoblast formation are shown in Figure 4. In Figures 1, 2, 3, 4, it is found that LPS could induce RAW264.7 cells into osteoclasts, and the LPS concentration in the range of 0–1 μg/mL showed a dose‐dependent promotion of osteoclast formation. The number of osteoclasts induced at LPS concentrations of 5 μg/mL or 10 μg/mL was not significantly different from that at an LPS concentration of 1 μg/mL. TRAP staining was consistent with this (Figures 2, 3, 4), and the concentration of 1 μg/mL was chosen for subsequent experiments. The inhibition effect on the differentiation of RAW264.7 cells into osteoclasts was investigated by selecting 1 μg/mL of LPS‐induced cells for 24 h, followed by the addition of different concentrations of drugs (betaine, itaconic acid, and fucose). It was found that in Figures 1, 2, 3, 4, betaine concentration in the range of 0–250 μM showed a dose‐dependent inhibitory relationship on the osteoclast formation, but the change in the osteoclast inhibitory effect was not significant in 250 μM, 500 μM, and 1000 μM. In Figures 1, 2, 3, 4, the concentration of itaconic acid in the range of 0–8 mM showed a dose‐dependent inhibition of osteoclast formation. In 4D‐1, a dose‐dependent inhibition of osteoclast formation was observed in the concentration range of 0–50 μg/mL, and the inhibitory effect on osteoclast formation was consistent at 50 μg/mL and 100 μg/mL of L‐fucose. All three screened active monomer components showed inhibitory effects on osteoclast formation.

The active ingredients in TCM could regulate bone formation and bone resorption, thus alleviating OP symptoms, with the inhibition of osteoclasts being an effective way to regulate the balance within the bone (Kenkre and Bassett 2018). The cellular results of the three screened active substances showed no cytotoxicity for LPS‐induced, while all inhibited osteoclast formation, indicating the potential of the screened substances to repair OP. Among them, betaine is an alkaloid that has been shown to have anti‐inflammatory, antifibrotic, and antiangiogenic properties and is often used in cosmetics, usually extracted from plants (Yajun et al. 2021). Studies on its extraction from Eucommia, Drynariae Rhizoma, and Fructus Psoraleae had not been reported and thus screened out as an active monomer in minor formula. Wang et al. explored the relationship between betaine and bone metabolism using intra‐ and extra‐human experiments (Yajun et al. 2021) and found that it reduced the thickness of the calcified cartilage and increased the expression level of lubricin, normalized the uncoupled subchondral bone remodeling, and significantly inhibited the number of osteoclast differentiation, which was the same as our results. In addition, it was found that betaine slowed down the progression of OA mainly by inhibiting overactivated osteoclast genesis and maintaining the microarchitecture of the subchondral bone, and we subsequently refer to the simplified three‐flavor minor formula for further validation of the therapeutic mechanism of OP.

L‐fucose was screened as a polysaccharide with certain immune activity and cancer inhibitory effect and also possessed certain potential to regulate bone formation and angiogenesis (Wang et al. 2021), which was mainly extracted from algae. In this experiment, L‐fucose, as one of the active substances in the simplified formula, was screened by bio‐chromatography, and it had a significant inhibitory effect on the formation of osteoclasts, which indicated that it could be used as one of the substances in the simplified formula. Itaconic acid was found to be an anti‐inflammatory metabolite related to aging, and its inhibitory effects on osteoclast differentiation and activation, as well as its rescuing effect in an animal model of LPS‐induced inflammatory bone loss (Wang et al. 2022), were observed. The cellular activity of our screened itaconic acid was found to have a significant effect on the inhibition of osteoclast formation, and thus it could be used as a monomeric active substance for the future treatment of OP as a simplified version. In conclusion, the cellular activity and osteoclast formation inhibition experiments showed that our screened active monomer had a significant effect on osteoclast formation inhibition, and the screened active monomer provided a theoretical basis for the simplification.

### 3.4 Results of Active Monomeric Substances on Inflammatory Expression in Osteoblasts

**FIGURE 5:**
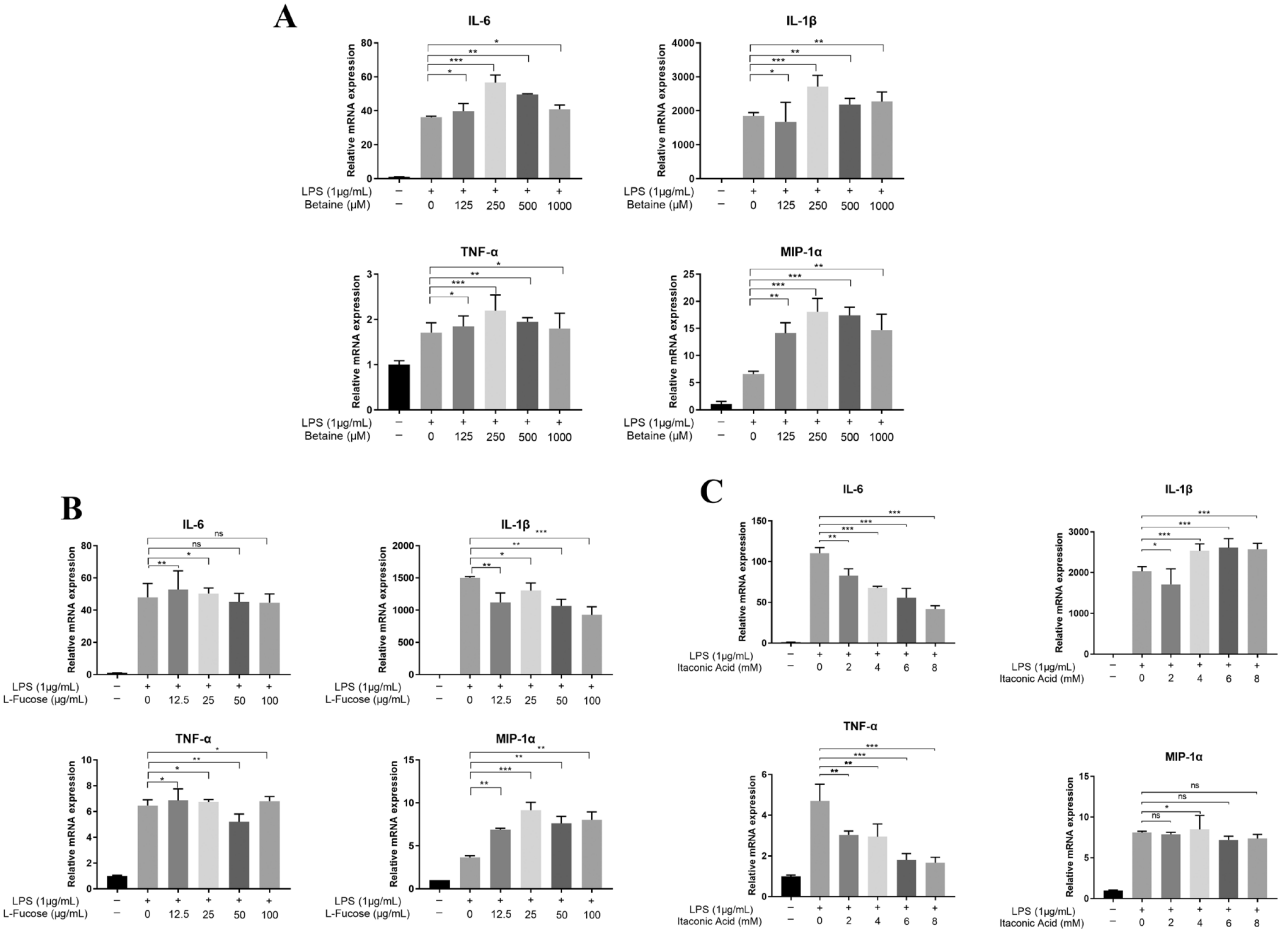
Effects of active monomeric substances on the inflammatory factor expression in osteoclasts in vitro. A. Effects of different concentrations of betaine on 1 μg/mL of LPS‐induced inflammation‐associated factors such as TNF‐α, IL‐6, IL‐1β, and MIP‐1α in osteoclasts. B. Effects of different concentrations of L‐fucose on 1 μg/mL of LPS‐induced inflammation‐associated factors such as TNF‐α, IL‐6, IL‐1β, and MIP‐1α in osteoclasts. C. Effects of different concentrations of itaconic acid on 1 μg/mL of LPS‐induced inflammation‐related factors TNF‐α, IL‐6, IL‐1β, and MIP‐1α in osteoblasts. *p < 0.05 as denoted by a bar, **p < 0.01 as denoted by a bar, ***p < 0.001 as denoted by a bar.

In order to explore more the specific effects of the screened active substances on osteoclast formation, we screened the osteoclastic inflammatory factors (TNF‐α, IL‐6, IL‐1β, and MIP‐1α) as discriminatory objects for osteoclast inflammation expression experiments, and the specific experimental results are shown in Figure 5. In Figure 5, a significant increase in the mRNA expression of intracellular inflammatory factors was observed after the induction of RAW264.7 cells with LPS, which indicated that the inflammatory model was successfully created. In Figure 5A, betaine did not exert a good inhibitory effect on the expression of inflammatory factors, but a trend of inhibitory effect in IL‐1β was exerted when betaine concentrations were up to 12.5 μM. In Figure 5B, after LPS induction of RAW264.7 cells, L‐fucose concentrations at 25–50 μg/mL exerted a trend of inhibitory effect on the expression of inflammatory factors TNF‐α, IL‐6, and IL‐1β but did not provide a good inhibitory effect on MIP‐1α. In Figure 5C, after the induction of RAW264.7 cells with 1 μg/mL of LPS, itaconic acid showed inhibitory effects on TNF‐α and IL‐6 within all concentrations and on IL‐1β at 2 mM, but all concentrations did not show good inhibitory effects on the expression of MIP‐1α.

When OP occurred in the body, the inflammatory activity of osteoclasts would be enhanced, and with the help of the LPS mediated the model of osteoclast differentiation and activity regulation in the inflammatory bone disease, which could help in clarifying the drug activity pattern (Kenkre and Bassett 2018; Tański, Kosiorowska, and Szymańska‐Chabowska 2021). The osteolytic effect induced by LPS was mainly caused by stimulating macrophages, fibroblasts, osteoblasts, and T lymphocytes to secrete large amounts of inflammatory cytokines such as TNF‐α, IL‐6, and IL‐1β (Amarasekara et al. 2018). In addition, inflammatory cytokines such as TNF‐α, IL‐6, and IL‐1β had been shown to induce the production of osteoclast precursor cells, which in turn could accelerate the formation of OP (Hasegawa et al. 2019). Therefore, the study of active monomeric components in TCM and osteoclast differentiation inflammation–related expression was one of the effective protocols to verify whether they had anti‐OP ability. It had been found in the experiments in Section 3.3 that the active substances in the three small formulas, which we simplified with the help of histological screening, all had an inhibitory effect on osteoclast formation, and in Section 3.4, the three active substances played a certain inhibitory effect on the expression of inflammatory factors. Betaine was found to alleviate orthopedic diseases primarily by inhibiting osteoclast genesis in vitro through the inhibition of reactive oxygen species (ROS) production and subsequent mitogen‐activated protein kinase (MAPK) signaling (Yajun et al. 2021). Its inhibition of inflammatory factors was not significant, and our experimental results in osteoblasts successfully modeled for inflammation showed that the inhibition was also not significant, similarly for L‐fucose. As mentioned above, itaconic acid as an inflammatory metabolite inhibits osteoporotic inflammation by suppressing the activation of NF‐κB and MAPK pathways through the inhibition of phosphorylation of IKKs, IκBα, P65, JNK, and P38, which in turn significantly inhibits the inflammatory expression (Wang et al. 2022). The results revealed that itaconic acid significantly reduced the expression of TNF‐α and IL‐6 in LPS‐stimulated mouse RAW264.7 macrophages, which indicated that the protective effect of itaconic acid on LPS‐stimulated bone resorption was partly attributed to its inhibition of proinflammatory cytokine production. This indicated that itaconic acid may have a specific target or mechanism in inhibiting the expression of inflammatory factors; we will add more relevant research data in the future research. The study of the active monomer and LPS‐mediated osteoclast‐associated inflammatory expression provided a significant insight into the pattern of bone metabolism in TCM treatment and also provided a basis for new directions to simplify the treatment of OP with the three small formulas.

## 4 Conclusions

Overall, we have performed the first separation, analysis, and bioactivity screening of the effective anti‐OP chemical components in F–E–D by solid‐phase bio‐cell chromatography and HPLC–MS, and finally selected betaine, L‐fucose, and itaconic acid as potential active candidate compound monomers for interaction with osteoblasts–osteoclasts. In terms of cellular validation, we found that the screened active ingredient monomers inhibited osteoclast formation and suppressed the expression of proinflammatory cytokines, suggesting that the screened active candidate compound monomers of F–E–D might be effective candidates for OP treatment. This study can provide the pharmacodynamic substance basis and relevant pharmacological data for the in‐depth development and utilization of F–E–D, as well as provide an effective method for the study of pharmacodynamic substances of TCM with unclear active ingredients and promote the modernization of TCM.

## Author Contributions

**Liming Zhu:** data curation (equal), investigation (equal). **Yeqing Wang:** investigation (equal), methodology (supporting). **Zhongxin Zhu:** project administration (equal), writing – original draft (equal). **Xiaocong Yao:** funding acquisition (lead), writing – original draft (equal). **Ruijuan Zhang:** data curation (equal), methodology (equal). **Yujie Xia:** methodology (lead), project administration (equal), writing – review and editing (equal). **Minbo Liu:** methodology (equal), project administration (equal), writing – review and editing (equal).

## Ethics Statement

The authors have nothing to report.

## Consent

The authors have nothing to report.

## Conflicts of Interest

The authors declare no conflicts of interest.

## Supporting information


Appendix S1.

Appendix S2.

## Data Availability

The datasets used to support the findings of this study are available from the corresponding author upon request.
